# S-1 Maintenance Therapy in Extensive Stage Small-Cell Lung Cancer—A Randomized Clinical Study

**DOI:** 10.1177/1073274820932004

**Published:** 2020-06-19

**Authors:** Keke Nie, Xiuhui Guo, Yunhong You, Xingjun Zhuang, Chunling Zhang, Youxin Ji

**Affiliations:** 1 Department of Oncology, Qingdao Central Hospital, Qingdao University, China; 2 Pingdu People’s Hospital, Qingdao, China; 3 Department of Oncology, PLA 971 Hospital, Qingdao City, China

**Keywords:** S-1, maintenance therapy, extensive-stage small-cell lung carcinoma, tumor regression rate

## Abstract

Small-cell lung cancer (SCLC) is a recalcitrant cancer for its dismal prognosis although extensive research had been done. Four to 6 cycles platinum-based chemotherapy is the mainstay treatment for the extensive-stage disease; but the role of maintenance treatment is not fully understood. This is a phase 2, open-label study. Patients with extensive-stage SCLC reaching an objective response or stable disease (SD) after induction chemotherapy were randomly assigned (1:1) with a minimization procedure. One group received oral S-1 and the other group received placebo as maintenance treatment until disease progression or unacceptable toxicities. The primary end point of this study was progression-free survival (PFS), and the secondary end points were overall survival (OS), response rates, and toxicities. This study was based on earlier work, the preliminary results was reported on 2019 ASCO annual meeting. A total of 89 patients were enrolled, of whom 45 received S-1 maintenance therapy and 44 received placebo. The median PFS and OS were 6.35 months and 10.82 months in the S-1 group, as compared to 5.98 months and 10.09 months in the placebo group. The PFS was 7.2 months and 5.3 months, and OS was 12.9 months and 10.9 months in patients with an objective response compared to in patients with SD after induction chemotherapy, respectively. S-1 maintenance therapy did not prolong PFS or OS in patients with extensive-stage SCLC; tumor regression rate was the prognostic factor of PFS or OS. Further research with novel agents in the maintenance setting is warranted.

## Introduction

Lung cancer is the leading cause of cancer death globally as well as in China,^1^ 10% to 15% of them are small-cell lung cancer (SCLC).^2,3^ About 80% patients of SCLC are in extensive stage at diagnosis, of them, the 5-year survival rate is only 1% to 2% even though tremendous studies on it in recent 3 decades.^1,4^ Four to 6 cycles chemotherapy with EP (cisplatin/carboplatin and etoposide) or IP (cisplatin/carboplatin and irinotecan) is the mainstay treatment for the extensive-stage SCLC; despite response rates of 60% to 70%, a median overall survival (OS) is approximately 10 months.^5,6^ Four to 6 cycles chemotherapy combined and/or maintained with programmed cell death-ligand 1 (PD-L1) monoclonal antibody (atezolizumab or durvalumab) treatment had very limited benefit on response rate, progression-free survival (PFS), and OS compared to chemotherapy alone.^7,8^ Therefore, to elicit a cost-effective maintenance agent for extensive-stage SCLC after chemotherapy is critical important.

S-1 (TS-1, Taiho Pharmaceutical Co) is a novel oral dihydropyrimidine dehydrogenase inhibitory fluoropyrimidine based on a biochemical modulation of 5-fluorouracil (5-FU), which was developed in 1990s for the treatment of gastric cancer. It contains tegafur (FF) and 2 types of enzyme inhibitor, 5-chloro-2, 4-dihydroxypyridine and potassium oxonate (Oxo), in a molar ratio of 1:0.4:1.^9,10^ In pharmacokinetic studies, S-1 showed high 5-FU concentration in blood for long periods of time. S-1 worked well in local advanced and metastatic gastric cancer and also showed high efficacy with tolerable toxicity in non-small cell lung cancer.^11^ How works and the roles of maintenance therapy of S-1 in SCLC are not fully understood. We carried out a preliminary study in patients with extensive-stage SCLC, to compare the efficiency and toxicities of S-1 maintenance therapy with observation. This study was retrospectively registered on ClinicalTrials.gov (NCT03769935) on December 10, 2018, and the preliminary results of this study were reported on 2019 ASCO annual meeting and published on *J Clin Oncol* 37, 2019 (suppl; abstr e20080).^12^

## Methods

### Study Design and Patient Selection

This is a 3-center, open-labeled, randomized study. Enrolled patients were histologically or cytologically confirmed stage IVSCLC by the International Association for the Study of Lung Cancer (IASLC) seventh edition,^13^ age 18 to 80 years old, with Eastern Cooperative Oncology Group (ECOG) performance status of 0 to 2 and treatment naive. Patients must have adequate bone marrow, renal, and hepatic function. Patients were required to have one or more evaluable target lesions which could be measured in one dimension according to Response Evaluation Criteria in Solid Tumors (RECIST) version 1.1.^14^ Central nervous system metastases at screen were excluded from the study.

**Figure 1.**
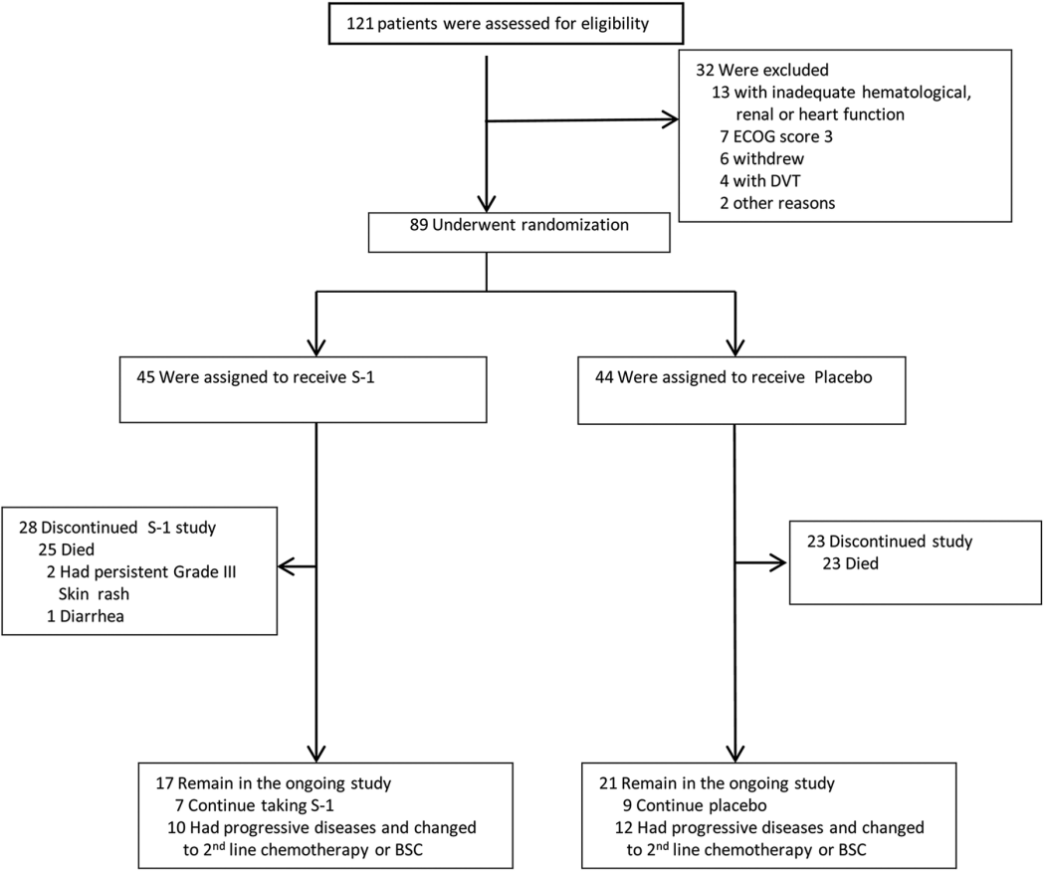
Trial profile. Data of cutoff date were November 30, 2018. Overall survival data were obtained on November 30, 2018.

Computerized randomization was done by center of the Qingdao Central Hospital, Qingdao University using Microsoft Excel 2007 formula and was dispensed to researchers case by case. When patient was qualified to the trial and informed consent was signed, the trial center of Qingdao Central Hospital would be informed and randomization would be done. Study group was received S-1 25 mg/m^2^ twice a day orally, and the other group was received placebo and regularly follow-up as control. Randomization was performed with dynamic balancing^15^ with respect to performance status, assessed using the World Health Organization performance scale measure activity, sex. S-1 treatment continued until: (1) the disease progression defined by RECIST version 1.1, (2) uncontrollable serious adverse effects or death, and (3) requested by patients or physician. On request was defined as physician’s request to stop, based on the patient’s condition was in dangerous if the trial continue. Dose adjustments and crossover were not allowed. Patients would be withdrawn from the study if they suffered intolerable drug-related toxicities (Figure 1).

Induction chemotherapy were initiated with EP regimen (etoposide 100 mg/m^2^ intravenous infusion on days 1, 2, 3 and cisplatin 75 mg/m^2^ or carboplatin AUC 5 intravenous infusion on day 1) or IP regimen (irinotecan 65 mg/m^2^ and cisplatin 30 mg/m^2^ intravenous infusion on day 1 and day 8); every 21 days a cycle for total 4 to 6 cycles. Patients enrolled this study must have reached complete response (CR), or partial response (PR), or stable disease (SD) per RECIST version 1.1 following completion of 4 to 6 cycles of induction therapy. All eligible patients were randomly assigned into 2 groups, in a 1:1 ratio. Imaging data were evaluated and reviewed by Centralized Independent Review Committee. One time to reduce 20% of dosage of EP or IP chemotherapy was permitted if patients acquired grade III to IV toxicity; but no further dose reduction was permitted. S-1 dosage adjustment was not allowed.

### Outcomes and Assessment

The primary end point of the study was PFS, measured from enrollment date to disease progression or death. The second end points were response rates, toxicities, and OS. Response rates were assessed using RECIST version 1.1, observed during trial period, classified into: CR (disappearance of tumor lesions), PR (a decrease of at least 30% in the sum of tumor lesions sizes), stable disease (steady state of disease), or progressive disease (an increase ≥20% in the sum of tumor lesions sizes). All adverse events were recorded and classified by grade according to the National Cancer Institute Common Terminology Criteria for Adverse Events version 4.0.^16^ The quality of life was assessed by KPS and recorded as apparently improved (increase in KPS by ≥20 posttreatment), improved (KPS score increase ≥10), stable (no apparent change in KPS score), and reduced (KPS score decline ≥10).

Tumor measurements were performed at screening and every 6 weeks thereafter. The results were reviewed by Independent Review Committee of Qingdao Central Hospital. Patients’ compliance, treatment safety, and side effects were accessed at each check point on every 6 weeks.

### Statistical Analysis

This was a superiority study. A sample size of 156 patients was calculated for each group, a type I error of 0.05 (one-side) and 80% power of test, and a 0.5 coefficient of variability at a 1:1 sample ratio of the 2 groups was assumed by us. The anticipated dropout rate was 10% and the actual value of coefficient of variability was likely to over 0.5, the optimum sample size would be 196 patients per group if hazard ratio (HR) was close to 0.85 in this study. The superiority would be established if the upper limit of 95% CI for the HR of S-1 versus placebo was less than 0.85 in the full analysis set.

Based on the Cox proportional hazards model, and taking into account the influence of gender (male or female), ECOG performance status score (0 vs 1 vs 2, and chemotherapy regimens, HR and 95% CI) were calculated in the full analysis population. Progression-free survival and OS curves were analyzed using Sigmaplot 11 (Systat software Inc) Kaplan-Meier log-rank test, and the HRs using Cox proportional hazards model in the intention-to-treat principle to compare the S-1 maintenance treatment group with the placebo group. Interim analyses and report were planned at 12 months of the study in the protocol. Early stopping of the study was allowed if the interim analyses data indicated that the S-1 group was clearly superior or inferior to the placebo group. Ethical board would be informed if there was unexpected adverse event happening or protocol modified.

The response rate, symptom reduction, and treatment-related adverse events were assessed with Fisher exact test (all randomly assigned patients received at least one dose of study drug).

## Results

A total of 121 patients were screened and 89 of them were enrolled into the study from January 2017 to November 2018. Thirty-two patients were excluded from the study because they did not meet the inclusion standard. The 2 groups were well balanced, with 45 patients were randomly assigned to the S-1 group, 44 patients were in the control group (Figure 1).

All recruited patients were Chinese. We analyzed enrolled patients’ age, performance status, according to centers with Fisher exact text, there was no significant difference. All patients were in stage IV according to the IASLC seventh edition staging system. A total of 71.9% of patients (64 of 89) were men, the median age was 67-year-old. All patients’ ECOG performance status scores were range in 0 to 2. The most common metastatic or recurrent sites after induction chemotherapy were lung, lymph nodes, adrenal gland, liver, and brain. The median follow-up time was 13.0 months and the last follow-up date was November 30, 2018 (Table 1).

**Table 1.** Baseline Characteristics of All Enrolled Patients.

Characteristic	S-1 group (N = 45)	Placebo group (N = 44)	*P* value
Median age (range)—years	68 (45-80)	65 (49-80)	
Age group, no (%)			.966
<65 years	16 (35.6)	14 (31.8)	
≥65 years	29 (64.4)	30 (68.2)	
Sex, no (%)			.974
Male	33 (73.3)	31 (70.5)	
Female	12 (26.7)	13 (29.5)	
Smoking status, no. (%)			.979
Never	13 (28.9)	12 (27.3)	
Former	1 (2.2)	2 (4.5)	
Current	31 (68.9)	30 (68.2)	
Histology, no. (%)			.988
Small-cell carcinoma	43 (95.6)	43 (97.7)	
Mixed cell carcinoma	2 (4.4)	1 (2.3)	
Chemotherapy regimen, no (%)			.975
EP	31 (68.9)	33 (75%)	
IP	14 (31.1)	11 (15.7)	
Previous chemotherapy response, no (%)			.984
CR + PR	39 (86.7)	36 (81.8)	
SD	6 (13.3)	8 (18.2)	
EP or IP dose reduction	8 (17.8)	8 (18.2)	NA
Previous PCI, no (%)	27 (60.0)	27 (61.4)	NA
Metastatic or recurrent site, no (%)			.776
Lung	23 (51.1)	21 (47.7)	
Liver	8 (17.8)	9 (20.5)	
Adrenal gland	11 (24.4)	10 (22.7)	
Lymph nodes	13 (28.9)	11 (25.0)	
Brain	5 (11.1)	5 (11.4)	
Other	9 (20.0)	7 (15.9)	
ECOG performance status, no (%)			.983
0	5 (11.1)	5 (11.4)	
1	35 (77.8)	36 (81.8)	
2	5 (11.1)	3 (6.8)	

Abbreviation: CR, complete response; ECOG, Eastern Cooperative Oncology Group performance status score, range from 0 to 5, with higher score reflecting greater disability; EP, Etoposide and cisplatin; IP, Irinotecan and cisplatin; PCI, prophylactic cranial irradiation; PR, partial response; SD, stable disease.

**Figure 2.**
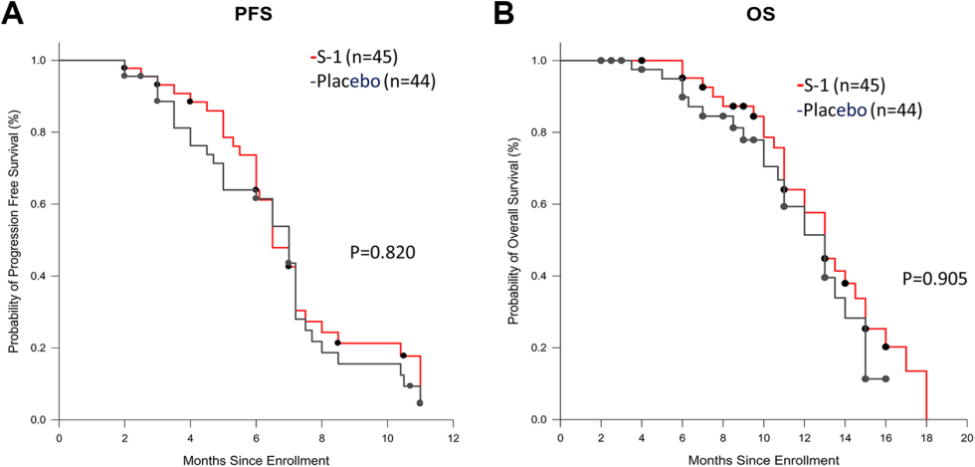
Kaplan-Meier analysis of progression-free survival (A) and overall survival (B) in the full analysis set. HR indicates hazard ratio.

Among 89 patients, of whom 45 received S-1 maintenance therapy and 44 received placebo. The median PFS was 6.35 months in the S-1 group, as compared to 5.98 months in the placebo group (HR for progression in the S-1 group, 1.057; 95% CI: 0.656-1.707; *P* = .820; Figure 2A). The median OS was 10.82 months in the S-1 group, as compared to 10.09 months in the placebo group (HR for death in the S-1 group, 0.860; 95% CI: 0.374-1.617; *P* = .905; Figure 2B).

**Figure 3.**
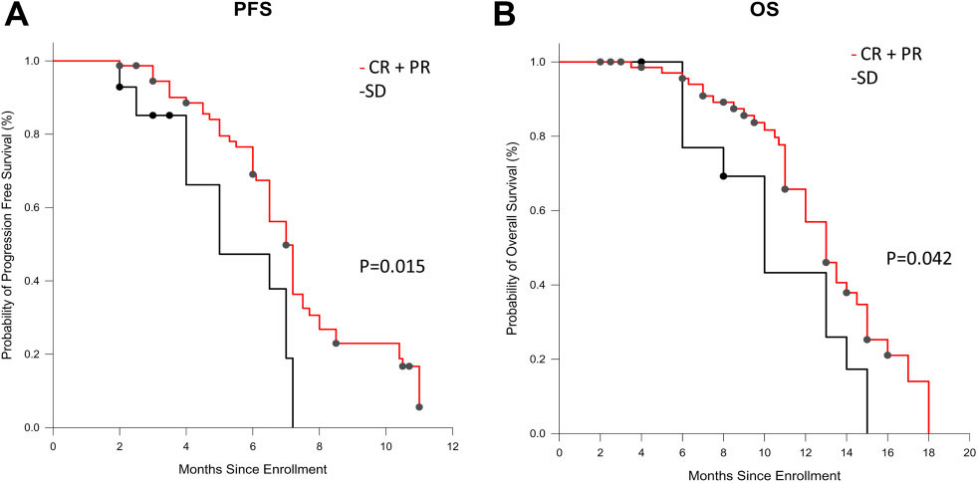
Kaplan-Meier analysis of progression-free survival (A) and overall survival (B) according to tumor response during induction chemotherapy. HR indicates hazard ratio.

Tumor regression rate influenced patients’ survival. The PFS and OS in patients with CR or PR after induction chemotherapy were 7.2 months and 12.9 months compared to 5.3 months and 10.9 months in patients with SD, respectively (Figure 3A-B).

The main adverse effects of the S-1 group were hand and foot syndrome, including rash or acne, dry skin, and darkened skin color, the incidence of which was much higher in the S-1 group than the incidence in the placebo group, and there was a significant difference. The anorexia, vomiting, alopecia and fatigue were higher in the S-1 group compared with the placebo group; and the hematological toxicities were neutropenia, thrombocytopenia, and anemia; however, there was no significant difference between the 2 groups. One patient had interstitial lung disease (ILD) in each group, cancer cell infiltrated ILD was diagnosed by radiologists and oncologists. There was no treatment-related death in both groups (Table 2). Qualities of life of patients were also accessed between the 2 groups at baseline and at last follow-up, there was no significant difference.

**Table 2.** Summary of Adverse Events.^a^

Adverse events	S-1 group (N = 45)			Placebo group (N = 44)			*P* value
	Grade 1 or 2	Grade 3 or 4	Grade 5	Grade 1 or 2	Grade 3 or 4	Grade 5	
Number (percent)							
Rash or acne	26 (57.8)	6 (13.3)	0 (0)	1 (2.3)	0 (0)	0 (0)	.002
Diarrhea	7 (15.6)	1 (2.2)	0 (0)	2 (4.5)	0 (0)	0 (0)	.085
Dry skin	11 (24.4)	0 (0)	0 (0)	1 (2.3)	0 (0)	0 (0)	.008
Darkened skin color	29 (64.4)	1 (2.2)	0 (0)	0 (0)	0 (0)	0 (0)	NA
Anorexia	16 (35.6)	0 (0)	0 (0)	2 (4.5)	0 (0)	0 (0)	.015
Nausea	8 (17.8)	0 (0)	0 (0)	2 (4.5)	0 (0)	0 (0)	.059
Vomiting	3 (6.7)	1 (2.2)	0 (0)	0 (0)	0 (0)	0 (0)	NA
Constipation	6 (13.3)	1 (2.2)	0 (0)	1 (2.3)	0 (0)	0 (0)	.031
Alopecia	23 (51.1)	9 (20.0)	0 (0)	21 (47.7)	8 (18.2)	0 (0)	.455
Neutropenia	8 (17.8)	0 (0)	0 (0)	3 (6.8)	0 (0)	0 (0)	.123
Thrombocytopenia	6 (13.3)	3 (6.7)	0 (0)	2 (4.5)	0 (0)	0 (0)	.125
Anemia	5 (11.1)	0 (0)	0 (0)	2 (4.5)	0 (0)	0 (0)	.138
Fatigue	11 (24.4)		0 (0)	2 (4.5)	1 (2.3)	0 (0)	.030
ILD	1 (2.2)	0 (0)	0 (0)	1 (2.3)	0 (0)	0 (0)	.500

Abbreviation: ILD, interstitial lung disease.

^a^ The date of data cutoff was November 30, 2018. Multiple occurrences of the same adverse events in one patient were counted once at the highest grade for the preferred term. The incidence of treatment-related adverse events associated with any component of the trial regimen was shown.

## Discussion

Four to 6 cycles EP or IP chemotherapy is the mainstay treatment for the extensive-stage SCLC; despite response rates of 60% to 70%, a median OS was approximately 10 months.^5,6^ The role of maintenance treatment is not fully understood and the prognosis remains poor. Many trials had evaluated maintenance therapy in extensive-stage SCLC, and most of them had failed in significant in clinical outcomes.^4,6,17,18^ The GOIRC-AIFA FARM6PMFJM-phase III trial indicated bevacizumab plus cisplatin and etoposide in the first-line treatment of extensive-stage SCLC and then bevacizumab maintenance treatment had a statistically significant improvement in PFS, which, however, did not translate into a statistically significant increase in OS.^3^ Another clinical trial showed that maintenance apatinib was safe and achieved encouraging PFS and OS in extensive-stage SCLC^2^; however, it was a single-arm retrospective study with fewer cases. The IMpower133 Clinical Trial, the median PFS was 5.2 months in the atezolizumab group and 4.3 months in the placebo group, respectively, which has been the only trial translating PFS into a statistically significant increasing in OS, revealed that atezolizumab plus carboplatin and etoposide resulting in significantly longer PFS and OS than chemotherapy.^7^ The CASPIAN trial was the newest clinical trial, in the immunotherapy group patients received up to 4 cycles of platinum–etoposide plus durvalumab followed by maintenance durvalumab every 4 weeks, which showed a significant improvement in OS, with an HR of 0.73. Median OS was 13·0 months in the immunotherapy group versus 10·3 months in the platinum–etoposide group.^8^ So the standard chemotherapy (EP) plus PD-L1 inhibitor (atezolizumab or durvalumab) then flowed by PD-L1 inhibitor maintenance treatment was recommended for extensive-stage SCLC; however, the median OS was prolonged just only about 2 months compared with the standard chemotherapy.^7-8^ Whether bevacizumab with PD-L1 inhibitor plus chemotherapy would be an optimal combination in extensive-stage SCLC needs more clinical trials.

S-1 at a dose of 80 mg/m^2^/d orally for 14 days every 3 weeks was effective and tolerable in non-SCLC.^11,19^ Because of its low toxicity and easy administration, maintenance therapy of S-1 was performed for patients with extensive-stage SCLC who did not have disease progression after first-line treatment in our study. The main adverse effects of the S-1 group were hand and foot syndrome, including rash or acne, dry skin, and darkened skin color, the incidence of which was much higher in S-1 group than the incidence in the placebo group, which were mild to moderate and higher than the placebo group, and there was a significant difference. The anorexia, vomiting, alopecia, and fatigue also higher in the S-1 group compared with the placebo group. The hematological toxicities were neutropenia, thrombocytopenia, and anemia, which were higher than the placebo group and were tolerated; however, there was no significant difference between the 2 groups.

In our study, the median PFS and OS in the S-1 group were 6.35 months and 10.82 months, and the median PFS and OS in the placebo group were 5.98 months and 10.09 months, respectively. There was no significant difference between the 2 groups. S-1 maintenance therapy did not prolong patients’ PFS or OS, but increased treatment-related adverse events; tumor regression rate was the main factor that influenced patients’ survival. The PFS and OS in patients with CR or PR after induction chemotherapy were 7.2 months and 12.9 months compared to 5.3 months and 10.9 months in patients with SD, respectively. Standard of first-line treatment with platinum-based chemotherapy for extensive-stage SCLC, a median OS was in range of 9 to 11 months,^20,21^ which was the same in our study.

## Conclusion

S-1 maintenance therapy in patients with extensive-stage SCLC after induction chemotherapy was safe but did not prolong PFS or OS. Tumor regression rate after induction therapy was the prognostic factor of PFS and OS. Further research with novel agents in the maintenance setting and maintenance therapy after second-line chemotherapy are needed in the future.

## Supplemental Material

Supplemental Material, CCX-19-0211.R1_-_Checklist - S-1 Maintenance Therapy in Extensive Stage Small-Cell Lung Cancer—A Randomized Clinical Study Click here for additional data file. Supplemental Material, CCX-19-0211.R1_-_Checklist for S-1 Maintenance Therapy in Extensive Stage Small-Cell Lung Cancer—A Randomized Clinical Study by Keke Nie, Xiuhui Guo, Yunhong You, Xingjun Zhuang, Chunling Zhang and Youxin Ji in Cancer Control

Supplemental Material, CCX-19-0211.R1_-_Registration - S-1 Maintenance Therapy in Extensive Stage Small-Cell Lung Cancer—A Randomized Clinical Study Click here for additional data file. Supplemental Material, CCX-19-0211.R1_-_Registration for S-1 Maintenance Therapy in Extensive Stage Small-Cell Lung Cancer—A Randomized Clinical Study by Keke Nie, Xiuhui Guo, Yunhong You, Xingjun Zhuang, Chunling Zhang and Youxin Ji in Cancer Control
