# Supplementary material for: S-1 Maintenance Therapy in Extensive Stage Small-Cell Lung Cancer—A Randomized Clinical Study
Source: Cancer Control. 2020 Jun 19;27(2):1073274820932004. doi: 10.1177/1073274820932004 (PMC7307401; doi:10.1177/1073274820932004)
Supplement: Supplemental Material, CCX-19-0211.R1_-_Registration - S-1 Maintenance Therapy in Extensive Stage Small-Cell Lung Cancer—A Randomized Clinical Study [file CCX-19-0211.R1_-_Registration.docx]

# Registry name; Preliminary Study of Maintenance Therapy for Patients With Extensive Stage of Small-cell Lung Cancer

**URL;** <https://clinicaltrials.gov/ct2/show/NCT03769935?term=youxin+ji&draw=2&rank=2>

Registration number; NCT 03769935
